# Laparoscopic Hernia Repair with the Extraperitoneal Approach versus Open Hernia Repair in Pediatric Inguinal Hernia: A Systematic Review and Meta-Analysis

**DOI:** 10.3390/jcm11020321

**Published:** 2022-01-10

**Authors:** Fu-Huan Huang, Po-Lung Cheng, Wen-Hsuan Hou, Yih-Cherng Duh

**Affiliations:** 1 Department of Biological Science and Technology, National Yang Ming Chiao Tung University, Hsinchu 300, Taiwan; 2 Division of Pediatric Surgery, Department of Surgery, Taipei Medical University Hospital, Taipei 110, Taiwan; 3 Department of Medical Education, Taipei Medical University Hospital, Taipei 110, Taiwan; b101105142@tmu.edu.tw; 4 Master Program in Long-Term Care and School of Gerontology Health Management, College of Nursing, Taipei Medical University, Taipei 110, Taiwan; houwh@tmu.edu.tw; 5 Department of Geriatrics and Gerontology, Taipei Medical University Hospital, Taipei 110, Taiwan; 6 Department of Physical Medicine and Rehabilitation, Taipei Medical University Hospital, Taipei 110, Taiwan; 7 Center of Evidence-Based Medicine, Department of Education, Taipei Medical University Hospital, Taipei 110, Taiwan; 8 Division of Pediatric Surgery, Department of Surgery, Hsinchu MacKay Memorial Hospital, Hsinchu 300, Taiwan; yicherng@gmail.com; 9 MacKay Junior College of Medicine, Nursing, and Management, New Taipei 252, Taiwan

**Keywords:** pediatric, inguinal hernia, laparoscopy, extraperitoneal, systemic review, meta-analysis

## Abstract

Objective: This systematic review and meta-analysis investigated the feasibility and effectiveness of laparoscopic hernia repair with the extraperitoneal approach in pediatric inguinal hernias. Summary Background Data: Inguinal hernia repair is the most common operation in pediatric surgical practice. Although open hernia repair (OHR) is a well-established procedure with good outcomes, studies have reported acceptable or even better outcomes of laparoscopic hernia repair with the extraperitoneal approach (LHRE). However, a meta-analysis comparing LHRE with OHR is lacking. Methods: PubMed, EMBASE, and Cochrane Library databases were searched for randomized controlled trials (RCTs) and comparative studies (prospective or retrospective). Outcomes were metachronous contralateral inguinal hernia (MCIH), hernia recurrence, surgical site infection, operation time, and hospitalization length. A meta-analysis was performed, and risk ratios (RR), weighted mean difference (WMD), and 95% confidence intervals (CI) were calculated using random-effects models. Results: Five RCTs and 21 comparative studies involving 24,479 patients were included. Lower MCIH incidence (RR: 0.11, 95% CI: 0.07 to 0.17; *p* < 0.00001) and a trend of shorter operation time (WMD: −11.90 min, 95% CI: −16.63 to −7.44; *p* < 0.00001) were found in the LHRE group. No significant differences in ipsilateral recurrence hernias, surgical site infection, and length of hospitalization were found between the groups. Conclusions: LHRE presented lower MCIH incidence and shorter operation times, with no increase in hernia recurrence, surgical site infection, or length of hospitalization. As more surgeons are increasingly becoming familiar with LHRE, LHRE would be a feasible and effective choice for pediatric inguinal hernia repair.

## 1. Introduction

Inguinal hernia is a common disorder in childhood that requires surgical repair [1]. Children who do not undergo repair are at a risk of incarceration, which occurs in approximately 3–16% of children with inguinal hernia [2,3]. Such surgical repair aims for the closure of the patent processus vaginalis (PPV) to be as high as possible. Open hernia repair (OHR) has been used with high success rates and low risks of complications [4], but the development of metachronous contralateral inguinal hernia (MCIH) is a challenge after unilateral OHR [5,6].

Laparoscopic hernia repair (LHR) became popular two decades after the first operation was performed by Montupet in 1993 [7]. The Evidence-Based Review Committee of the International Pediatric Endosurgery Group (IPEG) concluded that in comparison with OHR, LHR presented the advantages of shorter operation times in bilateral inguinal hernia repair and lower postoperative complications [8]. LHR can be achieved using the intraperitoneal or extraperitoneal approach. LHR with the intraperitoneal approach (LHRI) involves the application of two to three ports to enter the intraperitoneal space and perform the intracorporeal closure of the PPV, whereas LHR with the extraperitoneal approach (LHRE) involves the percutaneous introduction of suture material followed by the extracorporeal closure of the PPV. Laparoscopic percutaneous extraperitoneal closure (LPEC) and percutaneous internal ring suturing (PIRS) are the most common techniques of LHRE [9,10].

Two recent meta-analyses comparing the LHR with OHR reported inconsistent results in terms of LHR’s superiority [11,12]. Although Dreuning et al. concluded that LHR is not superior to OHR in terms of operation times in the case of unilateral inguinal hernia, a subgroup analysis in their study revealed the superiority of LHRE for both unilateral and bilateral hernia repair [11]. Kantor et al. implied that LHR presented advantages in terms of reduced complications and prevention of MCIH [12]. A review from IPEG found no high-level evidence indicating which type of LHR could be the best choice [8]. In addition to systematic reviews and meta-analyses, clinical trials and retrospective comparative studies have also been published more recently, and an updated meta-analysis could be necessary and reasonable. Therefore, the present study conducted a systematic review and meta-analysis of the superiority and feasibility of LHRE compared with OHR.

## 2. Materials and Methods

### 2.1. Selection Criteria

Studies that compared the outcomes of LHRE with those of OHR in pediatric patients who underwent inguinal hernia and were scheduled for surgery were included. Randomized controlled trials (RCTs) or comparative studies (prospective or retrospective) were also included. All included studies were required to clearly define the laparoscopic surgery and open surgery protocols. Exclusion was made for studies that compared LHRI with other surgery types; that only compared different laparoscopic surgery types; that only provided operation numbers rather than patient numbers; that were case reports, conference abstracts, reviews, systemic reviews, or meta-analyses; and those including patients aged over 18 years old.

### 2.2. Search Strategy

Records were identified through a search of the PubMed, EMBASE, and Cochrane Library databases. The following search headings were used: pediatric (laparoscopic OR open) AND hernia repair. These terms were also searched in the full text of the studies. The “related articles” function was used to search for more articles, and all the retrieved abstracts, studies, and citations were reviewed. Additional articles were identified through a manual search of the references and through experts in the field. No language limitation was applied. The final search was conducted on 1 July 2021. This systematic review has been accepted by PROSPERO, an online international prospective register of systematic reviews curated by the National Health Service (registration number: CRD42021229360).

### 2.3. Study Selection and Data Extraction

Two reviewers (PLC and HFH) independently screened the titles, abstracts, and full texts according to the selection criteria. Two reviewers (PLC and HFH) independently extracted the following information from each trial: first author, year of publication, study population characteristics, study design, inclusion and exclusion criteria, laparoscopic techniques, complications, and preoperative and postoperative parameters. The individually recorded decisions of the two reviewers were compared, and any disagreement was resolved by a third reviewer (HWH).

### 2.4. Outcome Measures

The primary outcome was MCIH incidence. The secondary outcomes were the incidence of an ipsilateral recurrence hernia, surgical site infection, operation time, and the length of hospitalization.

### 2.5. Methodological Quality Appraisal

Two reviewers (PLC and HFH) independently assessed the methodological quality of each RCT using the revised tool for assessing the risk of bias (RoB 2.0) and then assessed the quality of each comparative study using the Risk of Bias in Nonrandomized Studies of Interventions (ROBINS-I). In RoB 2.0, five domains of bias, namely bias arising from the randomization process, intended intervention, missing outcome data, outcome measurement, and selection of reported results, were assessed. Each trial was coded in terms of overall risk of bias, the risk of bias covers various aspects, and its score is that of the aspect carrying the greatest risk of bias [13]. In ROBINS-I, three main domains of bias, namely preintervention, intervention, and postintervention bias, were assessed. In each domain, different measurements were made, and an overall risk of bias was awarded [14]. The quality assessments are presented in Tables S1 and S2. All studies were included for statistic analysis regardless of bias assessment.

### 2.6. Statistical Analysis

The meta-analysis was performed according to recommendations by the Cochrane handbook [13] and fully compliant with the PRISMA (Preferred Reporting Items for Systematic Reviews and Meta-Analyses) and AMSTAR (Assessing the methodological quality of systematic reviews) Guidelines [15]. Statistical analyses were performed using the statistical program Review Manager, version 5.3 (Cochrane Collaboration, Oxford, UK). The effect sizes of dichotomous outcomes were reported as risks ratios (RR), and the weight mean differences (WMD) were reported for continuous outcomes. The precision of the effect size was reported in terms of the 95% confidence interval (CI). The standard deviation (SD) was calculated using the provided CI limits, standard errors, or interquartile ranges [16]. A pooled estimate of the RR was calculated using the DerSimonian and Laird random-effects model [17]. Two-sided *p*-values of <0.05 were considered statistically significant. If an RCT included more than two treatment arms, all data were used, as appropriate, without the repeated use of any component. To establish whether the results of the studies were consistent, we investigated statistical heterogeneity by evaluating *I*^2^ statistics; *I*^2^ values >75%, >50%, and <25% indicated high, moderate, and low heterogeneity, respectively. The proportion of the total outcome variability that was attributable to variability across the studies was quantified in terms of *I*^2^. If the heterogeneity was high, we conducted subgroup analysis and sensitivity analysis to investigate the heterogeneity.

## 3. Results

### 3.1. Characteristics of Studies

**Figure 1:**
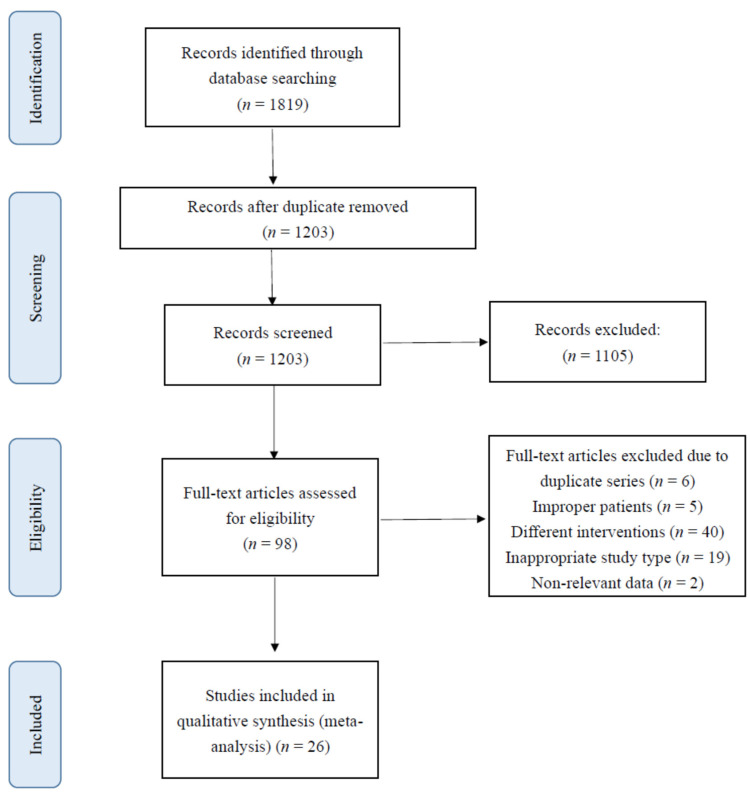
PRISMA flowchart.

Figure 1 present the flow of how studies were selected. In the initial search, we identified 106 citations from the Cochrane database, 768 citations from PubMed, 942 citations from EMBASE, and 3 other citations from the references of studies. We excluded 616 duplications and 1105 unrelated citations through the screening of titles and abstracts. After screening the full texts of the retrieved records, we further excluded 6 duplicate series, 5 citations for incorrect patients, 40 citations unrelated to the intervention in our study, 19 incorrect study design types, and 2 non-relevant data. Finally, 5 RCTs and 21 comparative studies (19 retrospective cohort and 2 prospective cohort) were included in the meta-analysis.

The characteristics of each included study are shown in Table 1 and Table 2. The studies were published between 2009 and 2021, with sample sizes ranging from 32 to 5393. The 26 included studies comprised 24,479 patients undergoing inguinal hernia repair, with 12,664 patients receiving LHRE and 11,815 receiving OHR. All patients were diagnosed with inguinal hernia, indirect inguinal hernia, incarcerated inguinal hernia, or hydrocele. Both the LHRE and OHR groups comprised patients with either unilateral hernia or bilateral hernia. The patients were infants and children below 18 years of age. The length of follow-up among patients was fairly variable, ranging from 1 month to several years. As many studies only included ranges of follow-up periods and no individual values, we were unable to calculate a median follow-up duration. In the LHRE group, 9068 patients were diagnosed as having unilateral hernia preoperatively. However, only 5922 patients received unilateral hernia repair, and the remaining patients received bilateral repair due to contralateral PPV. One study was excluded from the calculation because the numbers of bilateral hernia repairs due to contralateral PPV could not be identified [18].

### 3.2. Metachronous Hernia

**Figure 2:**
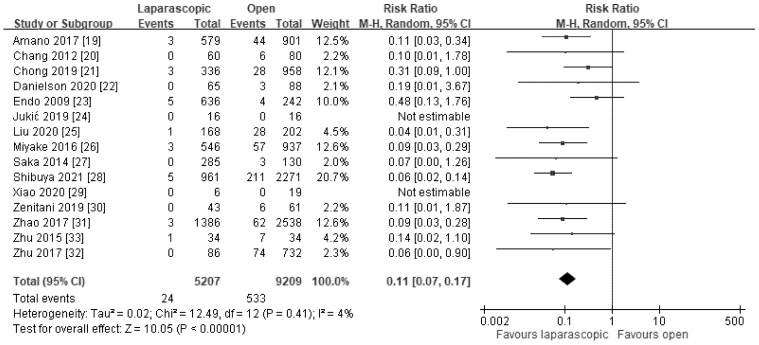
Forest plot of LHRE vs. OHR: incidence of metachronous hernia.

A total of 16 studies compared the incidence of metachronous hernia between the LHRE and OHR groups, and 15 of them with a total of 14,416 patients (5207 patients in the LHRE group and 9209 patients in the OHR group) with the diagnosis of unilateral inguinal hernia who received operation were assessed [21,23,24,25,26,27,29,32,33,36,37,40,41,42,43]. The pooled result indicated that LHRE resulted in a considerably lower incidence of metachronous hernia compared with OHR (RR: 0.11, 95% CI: 0.07 to 0.17; *p* < 0.00001; *I*^2^ = 4%; Figure 2).

### 3.3. Ipsilateral Recurrence Hernia

**Figure 3:**
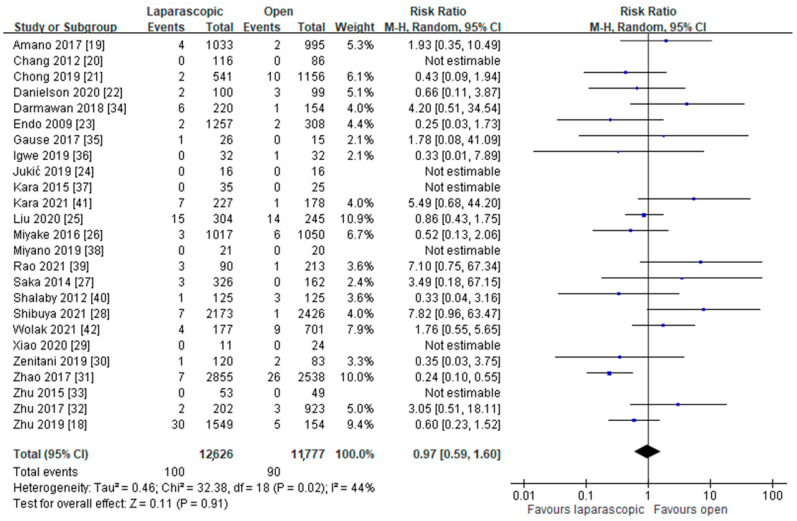
Forest plot of LHRE vs. OHR: incidence of ipsilateral recurrence hernia.

A total of 26 studies compared the incidence of ipsilateral recurrence hernia between the LHRE and OHR groups, and the data of 25 of them covered a total of 24,430 patients (12,626 patients in the LHRE group and 11,777 patients in the OHR group) were assessed [18,19,20,21,22,23,24,25,26,27,28,29,30,31,32,33,34,35,36,37,39,40,41,42,43]. The incidence of ipsilateral recurrence hernia did not differ between the LHRE and OHR groups (RR: 0.97, 95% CI: 0.59 to 1.60; *p* = 0.91; *I*^2^ = 44%; Figure 3).

### 3.4. Operation Time

**Figure 4:**
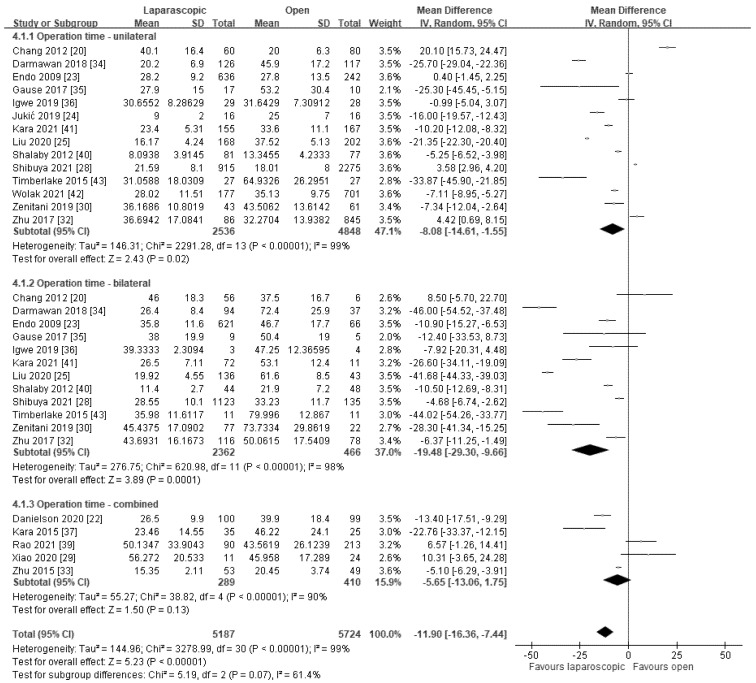
Forest plot of LHRE vs. OHR: operation time.

In total, 24 studies compared the operation time between the LHRE and OHR groups; 19 of these that clearly described the statistical data of unilateral hernia repair, bilateral hernia repair, or a combination of both were analyzed independently [19,20,21,22,23,25,27,28,29,30,31,32,35,37,39,40,41,42]. Overall, the pooled result revealed a significantly shorter operation time in the LHRE group than in the OHR group (WMD: −11.90 min, 95% CI: −16.36 to −7.44; *p* < 0.00001; *I*^2^ = 99%; Figure 4). Regarding the operation time for unilateral hernia repair, the pooled result revealed a shorter operation time in the LHRE group than in the OHR group (WMD: −8.08 min, 95% CI: −14.61 to −1.55; *p* = 0.02; *I*^2^ = 99%; Figure 4). Regarding the operation time for bilateral hernia repair, the pooled result revealed a significantly shorter operation time in the LHRE group than in the OHR group (WMD: −19.48 min, 95% CI: −29.30 to −9.66; *p* = 0.0001; *I*^2^ = 98%; Figure 4). The results of the five articles [24,26,33,34,36] not included in the pooled result are detailed in Table S3. As the heterogeneity was relatively high in terms of the operation time, we conducted a sensitivity analysis for further evaluation. Significant heterogeneity was still present even when we performed subgroup analyses, omitted studies with moderate to high risk of bias, or omitted one study in turn in the sensitivity analysis.

### 3.5. Surgical Site Infection

Fourteen studies compared the incidence of surgical site infection between the LHRE and OHR groups [19,20,21,24,26,27,30,31,32,33,34,35,39,40]. A total of 8574 patients (3750 patients in the LHRE group and 4123 patients in the OHR group) were assessed. The incidence of surgical site infection did not significantly differ between the LHRE and OHR groups (RR: 1.16, 95% CI: 0.53 to 2.52; *p* = 0.71; *I*^2^ = 44%).

### 3.6. Length of Hospitalization

Four studies compared the length of hospitalization between the LHRE and OHR groups [19,21,32,40]. A total of 657 patients (357 patients in the LHRE group and 300 patients in the OHR group) were assessed. The length of hospitalization was not significantly different between the LHRE and OHR groups (WMD: −0.04 days, 95% CI: −0.10 to 0.03; *p* = 0.25; *I*^2^ = 0%).

## 4. Discussion

In this systematic review and meta-analysis, we evaluated the superiority and feasibility of LHRE. We analyzed evidence from 5 RCTs and 21 comparative studies involving a total of 24,479 patients. Our study demonstrated that LHRE considerably reduced the incidence of MCIH compared with OHR. Moreover, LHRE was more beneficial in terms of the operation time, with a mean reduction of 11.90 min. Furthermore, the incidence of recurrence hernia, surgical site infection, and length of hospitalization did not differ between the LHRE and OHR groups. Therefore, LHRE might be a more feasible choice of minimally invasive operation for achieving favorable patient outcomes.

The incidence rate of MCIH has been reported to be approximately 6–12.3% [5,6]; Watanabe et al. reported that the incidence of MCIH was as low as 0.8% after a true negative finding on the other side during unilateral LHRE [38]. Previous meta-analyses have disagreed regarding whether LHR or OHR reduced the incidence of MCIH, and their results exhibited high heterogeneity (*I*^2^ = 52% and 78%) [13,14]. In our analysis, the incidence of MCIH was lower in the LHRE group with low heterogeneity (RR = 0.11, *p* < 0.00001; *I*^2^ = 4%). Our study revealed that extraperitoneal approaches had benefits in reducing the incidence of MCIH. Using a Taiwanese nationwide health insurance database, Lee et al. found that over two-thirds of patients who needed MCIH repair could receive a diagnosis within 3 years after initial repair; furthermore, over 90% of patients with the need for repair of MCIH could be identified within 5 years [5]. The insufficient follow-up period could have resulted in the underestimation of the incidence of MCIH. The superiority of LHRE in decreasing MCIH incidence may be attributed to the perioperative scrutiny of the contralateral inguinal ring through laparoscopy and the effective identification of the morphology of contralateral PPV for closure at the same time [44,45].

Dreuning et al. analyzed eight RCTs that compared the LHR with OHR and found no difference in operation times between the two in unilateral inguinal hernias [13]. However, their subgroup analysis revealed shorter operation times in the LHRE group compared with the OHR group in both unilateral and bilateral inguinal hernia repair. We conducted our meta-analysis on recent studies that included more patients who underwent LHRE than those who underwent OHR and observed that LHRE had shorter operation times in both unilateral and bilateral hernias. Our evaluation demonstrated that the operation time was reduced by 8.08 and 19.48 min in cases of unilateral hernia and bilateral operation, respectively. However, a high heterogeneity (*I*^2^ = 99%) remained even after sensitivity analysis. The operation time could have been mainly influenced by surgeons’ experience and the use of appropriate surgical devices. The learning curve of LHRE has been reported to be approximately 30 procedures for surgical trainees and 15 for experienced surgeons, and the operation time taken by an experienced surgeon for one procedure can be under 30 min [46,47]. Many devices are used for LHRE procedures, including a steel awl, spinal needles, a Reverdin needle, an Endoneedle, and specialized tools, but sufficient evidence regarding which device is superior is unavailable [8]. Therefore, LHRE may be superior in terms of operation time when the surgery is performed by experienced surgeons using devices familiar to them.

Previous studies have evaluated the incidence of surgical site infection [12,48,49]. Yang et al. reported that the incidence of surgical site infection did not differ between LHR and OHR [48]. Moreover, in a study by Kantor et al., OHR appeared to reduce the likelihood of wound infection, although most studies have reported nonsignificant differences in this regard, with one study’s results skewing the overall result [12]. By contrast, Esposito et al. demonstrated that OHR had a higher wound infection rate relative to LHR [48]. Generally, minimally invasive surgery is associated with lower risks of surgical site infection compared with conventional procedures [50]. The main incision site in LHR is the umbilicus, where it is difficult to remove all blots while maintaining aseptic conditions. One of the included trials even reported that in their institution, although the umbilicus was routinely cleaned meticulously before the operation, it remained unclean due to the blot [24]. Among the 14 included studies, the surgical site infection rates were 1.07% and 0.97%, respectively, for LHRE and OHR. However, our meta-analysis revealed no differences between the LHRE and OHR groups. Thus, LHRE does not increase the risk of surgical site infection.

Low heterogeneity was observed with respect to MCIH incidence, recurrence of the hernia, surgical site infection, and length of hospitalization, whereas extremely high heterogeneity was observed with respect to operation time. Therefore, the results of this meta-analysis should be interpreted with caution. The heterogeneity differed between our selected trials in the pooled results, which was attributable to various factors. First, the experience of performing laparoscopic operations varied among the surgeons. Laparoscopic surgery has a learning curve, and previous studies have indicated that a surgeon was required to have performed 35 laparoscopic operations to be considered experienced. Second, the characteristics of patients differed in the included studies. Sex, age, and hernia size might influence the outcome of the operation. Moreover, whether the operation was an emergency operation or elective operation may also influence the result.

Our study has several limitations. First, our study comprised both RCTs and comparative studies. The evidence strength may have been influenced because of the inclusion of retrospective studies. Second, the LHRE procedures differed between studies in terms of the number of ports, the device used for introducing suture material, and types of suture material, which might have increased the heterogeneity of our study results. Third, we had limited evidence about patients younger than 1-year-old who received LHRE, which might affect the generalizability of the results. Although we searched for studies with patients under 18 years old, the number of patients younger than 1-year-old was limited. Typically, laparoscopic surgery is difficult in neonates and small infants because their small abdominal capacity provides a small operative field. However, our study demonstrated that LHRE is a feasible and effective procedure for the pediatric population. In our meta-analysis, we included a retrospective study by Zenitani et al. that involved patients aged under 6 months. They reported that compared with OHR, LHRE was better in terms of the MCIH incidence and operation time [41]. Additional trials involving patients under 1 year of age should be conducted to further generalize the results.

In conclusion, LHRE is beneficial because it presents a lower incidence of MCIH and probably shorter operation times. Moreover, no difference in recurrence hernia, surgical site infection, or length of hospitalization was noted between the two methods. As the feasibility and effectiveness of LHRE has been verified, LHRE would be an appropriate choice for pediatric inguinal hernia repair.

## Figures and Tables

**Table 1:** Characteristics of randomized controlled trials.

Study	No. of Patients (% Male)	Age (Months), Mean ± SD	Preoperative Diagnosis No. of Unilateral (%)	Operation No. of Unilateral (%)	Follow-Up Period	Method of LHRE
Gause et al., 2017 [19]	L: 26 (73) O: 15 (80)	L: 11.5 ± 8.9 O: 5.5 ± 4.1	L: 23 (88) O: 14 (93)	L: 17 (65) O: 10 (67)	2 ± 2.7 y ^§^	laparoscopic subcutaneous endoscopically assisted ligation of the interring (SEAL technique)
Igwe et al., 2019 [20]	L: 32 (75) O: 32 (91)	L: 44 (2–156) ^＃^ O: 36 (2–168) ^＃^	L: 30 (94) O: 28 (88)	L: 29 (91) O: 28 (88)	3 m	laparoscopic needle assisted hernia repair (LNAR)
Jukić et al., 2019 [21]	L: 16 (100) O: 16 (100)	L: 60 (36–84) ^＃^ O: 48 (36–84) ^＃^	L: 16 (100) O: 16 (100)	L: 16 (100) O: 16 (100)	6 m	percutaneous internal ring suturing (PIRS)
Shalaby et al., 2012 [22]	L: 125 (70) ^◇^ O: 125 (74)	61.56 ± 28.32	L: 81 (65) O: 77 (62)	L: 81 (65) O: 77 (62)	24 m (16–30) ^＃^	laparoscopic assisted hernia repair (Reverdin Needle)
Zhu et al., 2015 [23]	L: 53 (66) O: 49 (73)	L: 23.5 (7–61) ^＃^ O: 21.5 (7–63) ^＃^	L: 42 (79) O: 34 (69)	L: 34 (64) O: 34 (69)	6 m	laparoscopic assisted extraperitoneal hernia sac high ligation

d: days; m: months; L: laparoscopic repair group; O: open repair group; LHRE: laparoscopic hernia repair with extra-peritoneal approach; SD: standard deviation; y: years. ^§^: mean ± SD, ^＃^: median (range). ^◇^: In addition to patients who presented with (i) unilateral inguinal hernia in obese children (L: 25; O: 28) and (ii) bilateral inguinal hernia (L: 44; O: 48), Shalaby et al. also included patients with (iii) recurrent inguinal hernia (L: 12; O: 15), (iv) inguinal hernia with an umbilical hernia (L: 18; O: 22), and (v) inguinal hernia with a questionable other side (L: 26; O: 12).

**Table 2:** Characteristics of comparative studies.

Study	No. of Patients (% Male)	Age (Months), Mean ± SD	Preoperative Diagnosis No. of Unilateral (%)	Operation No. of Unilateral (%)	Follow-Up Period	Method of LHRE
Amano et al., 2017 [24]	L: 1033 (47) O: 995 (64)	L: 49.0 ± 36.2 O: 48.8 ± 36.0	L: 959 (93) O: 901 (91)	L: 579 (56) O: 901 (91)	L: 29.1 ± 24.3 m ^§^ O: 49.3 ± 50.5 m ^§^	single-incision laparoscopic percutaneous extraperitoneal closure (SILPEC)
Chang et al., 2012 [25]	L: 116 (65) O: 86 (73)	L: 45.6 ± 49.2 O: 33.6 ± 34.8	L: 106 (91) O: 82 (95)	L: 60 (52) O: 80 (93)	L: 35.3 ± 6.8 m ^§^ O: 36.3 ± 7.7 m ^§^	single-port laparoscopic herniorrhaphy
Chong et al., 2019 [26]	L: 541 (74) O: 1156 (80)	detail below the table	L: 336 (62) O: 958 (83)	L: 336 (62) O: 958 (83)	L: 2.6 y O: 3.6 y	percutaneous internal ring suturing (PIRS)
Danielson et al., 2020 [27]	L: 100 (72) O: 99 (91)	L: 26 (1–165) ^＃^ O: 5 (0–164) ^＃^	L: 82 (82) O: 88 (89)	L: 65 (65) O: 88 (89)	6 m	percutaneous internal ring suturing (PIRS)
Darmawan et al., 2018 [28]	L: 220 (66) O: 154 (71)	L: 11 (0–192) ^＃^ O: 42.5 (1–210) ^＃^	L: 126 (57) O: 117 (76)	L: 126 (57) O: 117 (76)	5 y	transcutaneous trans-fixation ligature
Endo et al., 2009 [29]	L: 1257 (55) O: 308 (73)	L: 45.6 ± 34.8 O: 44.4 ± 38.4	L: 1201 (96) O: 294 (95)	L: 636 (51) O: 242 (79)	1–11 y ^＃^	laparoscopic patent processus vaginalis (PPV) closure
Kara et al., 2015 [30]	L: 35 (49) O: 25 (56)	L: 67.8 ± 44.76 O: 50.88 ± 47.76	L: 29 (83) O: 24 (96)	L: 29 (83) O: 24 (96)	at least 2–3 y	percutaneous internal ring suturing (PIRS)
Kara et al. ^†^ 2021 [31]	L: 227(63) O: 178(69)	L: 54.72 ± 44.88 O: 50.28 ± 40.08	L: 196 (86) O: 167 (94)	L: 155 (68) O: 167 (94)	L: 30.4 m O: 24.4 m	percutaneous internal ring suturing (PIRS)
Liu et al., 2020 [32]	L: 304 (89) O: 245 (89)	L: 15 (3–304) ^＃^ O: 15 (3–154) ^＃^	L: 249 (82) O: 202 (82)	L: 168 (55) O: 202 (82)	follow-up until September 2019	laparoscopic percutaneous extraperitoneal closure (two-hooked hernia needle)
Miyake et al., 2016 [33]	L: 1017 (55) O: 1050 (59)	L: 45 O: 44.64	L: 925 (91) O: 937 (89)	L: 546 (54) O: 937 (89)	L: 40 m O: 100 m	laparoscopic percutaneous extraperitoneal closure (LPEC)
Miyano et al.^†^ 2019 [34]	L: 21 (100) O: 20 (100)	L: 8.7 O: 8.6	L: 19 (90) O: 20 (100)	L: 19 (90) O: 20 (100)	at least 12 m	laparoscopic percutaneous extraperitoneal closure (LPEC)
Rao et al., 2021 [35]	L: 90 (77) O: 213 (77)	L: 5 (3.2–22.4) * O: 14 (3.2–64.5) *	L: 72 (80) O: 175 (82)	L: 49 (54) O: 155 (73)	L: 41.5 d (2–149.8) * O: 29 d (1–189) *	percutaneous internal ring suturing (PIRS)
Saka et al., 2014 [36]	L: 326 (38) O: 162 (86)	L: 55.8 ± 38.6 O: 38.3 ± 39.1	L: 291 (89) O: 145 (90)	L: 291 (89) O: 145 (90)	1 m	laparoscopic percutaneous extraperitoneal closure (LPEC)
Shibuya et al., 2021 [37]	L: 2173 O: 2426	L: 49.06 ± 36.06 ^▽^ O: 41.16 ± 33.4 ^▽^	L: 961 (44) O: 2271 (94)	L: 961 (44) O: 2271 (94)	Not mentioned	laparoscopic percutaneous extraperitoneal closure (LPEC)
Timberlake et al., 2015 [38]	L: 38 (90) O: 38 (95)	L: 21.5 (2–103) ^＃^ O: 23 (1–92) ^＃^	L: 27 (71) O: 27 (71)	L: 27 (71) O: 27 (71)	L: 51 d (37–113) ^＃^ O: 47 d (21–146) ^＃^	laparoscopic percutaneous hernia repair (LPHR)
Wolak et al., 2021 [39]	L: 177 (58) O: 701 (84)	detail below the table	L: 177 (100) O: 701 (100)	L: 177 (100) O: 701 (100)	detail below the table	percutaneous internal ring suturing (PIRS)
Xiao et al., 2020 [40]	L: 11 (0) O: 24 (0)	L: 71.4 ± 40.8 O: 67.6 ± 54.5	L: 6 (55) O: 19 (79)	L: 6 (55) O: 19 (79)	L: 14.5 ± 12.8 m ^§^ O: 71.3 ± 46.8 m ^§^	single-port laparoscopic percutaneous extraperitoneal closure
Zenitani et al., 2019 [41]	L: 120 (63) O: 83 (92)	L: 3 (0–5) ^＃^ O: 3 (1–5) ^＃^	L: 106 (88) O: 61 (73)	L: 43 (36) O: 61 (73)	L: 45.5 m (1–96) ^＃^ O: 73 m (1–95) ^＃^	laparoscopic percutaneous extraperitoneal closure (LPEC)
Zhao et al., 2017 [42]	L: 2855 (83) O: 2538 (84)	L: 29.28 ± 25.8 O: 29.4 ± 26.16	L: 2855 (100) O: 2538 (100)	L: 1386 (49) O: 2538 (100)	33.4 m (18–54) ^＃^	laparoscopic inguinal hernia repair
Zhu et al., 2017 [43]	L: 202 (73) O: 923 (83)	detail below the table	L: 153 (76) O: 845 (92)	L: 86 (43) O: 845 (92)	10.1 m (7–18)	laparoscopic hernia repair
Zhu et al., 2019 [18]	L: 1549 O: 154	17.2 ± 11.9	1421 (83)	1421 (83)	17.5 ± 4.3 m ^§^ follow-up until August 2018	laparoscopic percutaneous extraperitoneal closure (LPEC)

d: days; m: months; L: laparoscopic repair group; O: open repair group; LHRE: laparoscopic hernia repair with extra-peritoneal approach; SD: standard deviation; y: years. ^§^: mean ± SD; ^＃^: median (range); *: median (IQR). ^◇^: In addition to patients who presented with (i) unilateral inguinal hernia in obese children (L: 25; O: 28) and (ii) bilateral inguinal hernia (L: 44; O: 48), Shalaby et al. also included patients with (iii) recurrent inguinal hernia (L: 12; O: 15), (iv) inguinal hernia with umbilical hernia (L: 18; O: 22), and (v) inguinal hernia with questionable other side (L: 26; O: 12), ^▽^: The dataset for analysis of operative and anesthesia times, instead of all included patient, ^†^: Prospective cohort study (the rest: retrospective cohort study), Chong (2019) patient age: open unilateral group (147 patient < 1 yr, 245 patient 1–4 yr, 215 patient 5–9 yr, 78 patient 10–14 yr), open + explore (85 patient < 1 yr, 84 patient 1–4 yr, 85 patient 5–9 yr, 21 patient 10–14 yr), open bilateral (97 patient < 1 yr, 58 patient 1–4 yr, 31 patient 5–9 yr, 12 patient 10–14 yr), laparoscopic unilateral (125 patient < 1 yr, 79 patient 1–4 yr, 105 patient 5–9 yr, 27 patient 10–14 yr), laparoscopic bilateral (114 patient < 1 yr, 48 patient 1–4 yr, 35 patient 5–9 yr, 8 patient 10–14 yr). Wolak (2021) patient age: open male group (4.6 yr, range 0–18 yr), open female (6.34 yr, range 0–17 yr), laparoscopic male (5.36 yr, range 0–18 yr), laparoscopic female (5.47 yr, range 0–18 yr). Wolak (2021) follow-up period: open male (65.2 m, range 24–113 m), open female (62.3 m, range 25–98 m), laparoscopic male (68.5 m, range 25–100 m), laparoscopic female (64.1 m, range 26–99 m). Zhu (2017) patient age: laparoscopic group (202 patients with 42 patients < 1 yr); open group (923 patients with 104 patients < 1 yr).

## Data Availability

All data analyzed during this study are included in these published articles and their supplementary information files.

## Supplementary Materials

The following supporting information can be downloaded at: https://www.mdpi.com/article/10.3390/jcm11020321/s1, Table S1: Methodological quality assessment of randomized studies; Table S2: Methodological quality assessment of nonrandomized studies; Table S3: Studies excluded from the meta-analysis of operation time.
