# Supplementary material for: Laparoscopic Hernia Repair with the Extraperitoneal Approach versus Open Hernia Repair in Pediatric Inguinal Hernia: A Systematic Review and Meta-Analysis
Source: J Clin Med. 2022 Jan 10;11(2):321. doi: 10.3390/jcm11020321 (PMC8781267; doi:10.3390/jcm11020321)
Supplement: Supplementary file 1 [file jcm-11-00321-s001.zip › jcm-1514588-supplementary.pdf]

Journal of
Clinical Medicine

MDPI

Supplementary Material: Laparoscopic Hernia Repair with the Extraperitoneal Approach versus Open Hernia Repair in Pediatric Inguinal Hernia: A Systematic Review and Meta-Analysis

Fu-Huan Huang, Po-Lung Cheng, Wen-Hsuan Hou and Yih-Cherng Duh

Table S1. Methodological quality assessment of randomized studies.

Study	Bias Arising from the Randomisation Process	Bias Due to Deviations from Intended Interventions	Bias Due to Missing Outcome Data	Bias in Outcome Measurement	Bias in the Selection of the Reported Result	Overall Risk of Bias
Gause et al., 2017 [1]	Low risk	Low risk	Low risk	Low risk	Low risk	Low risk
Igwe et al., 2019 [2]	Low risk	Low risk	Low risk	Low risk	Low risk	Low risk
Jukić et al., 2019 [3]	Low risk	Low risk	Low risk	Low risk	Low risk	Low risk
Shalaby et al., 2012 [4]	Some concerns [a]	Low risk	Low risk	Low risk	Low risk	Some concerns
Zhu et al., 2015 [5]	Some concerns [a]	Some concerns [b]	Low risk	Some concerns [c]	Low risk	High risk

Methodological quality assessment was based on the Cochrane risk of bias tool (RoB 2.0). [a] Lack of information on the concealment of the allocation sequence. [b] Lack of information on blinding. [c] Lack of information on outcome assessor blinding.

Table S2. Methodological quality assessment of nonrandomized studies.

Study	Pre-Intervention		Intervention	Post-Intervention				Overall
	Bias due to Confounding	Bias in Selection	Bias in Classification of Interventions	Bias from Intended Interventions	Bias Due to Missing Data	Bias in Measurement of Outcomes	Bias in Selection of the Reported Result	Overall Bias
Amano et al., 2017 [6]	low	low	low	low	low	low	low	low
Chang et al., 2012 [7]	low	low	low	low	moderate [e]	low	moderate [f]	moderate
Chong et al., 2019 [8]	moderate [a]	low	low	low	low	low	low	moderate
Danielson et al., 2020 [9]	low	low	low	moderate [d]	low	low	low	moderate
Darmawan et al., 2018 [10]	low	moderate [b]	low	low	low	low	low	moderate
Endo et al., 2009 [11]	low	low	low	moderate [d]	low	low	low	moderate
Kara et al., 2015 [12]	low	low	low	low	low	low	low	low
Kara et al., 2021 [13]	low	low	low	low	low	low	low	low
Liu et al., 2020 [14]	low	low	low	low	low	low	low	low
Miyake et al., 2016 [15]	low	low	low	low	low	low	low	low
Miyano et al., 2019 [16]	low	low	low	low	low	low	low	low

Rao et al., 2021 [17]	low	low	low	low	low	low	low	low
Saka et al., 2014 [18]	low	low	low	low	low	low	low	low
Shibuya et al., 2021 [19]	low	low	low	low	low	low	low	low
Timberlake et al., 2015 [20]	low	low	low	low	low	low	low	low
Wolak et al., 2021 [21]	low	low	low	low	low	low	low	low
Xiao et al., 2020 [22]	low	low	low	low	low	low	low	low
Zenitani et al., 2019 [23]	low	moderate [c]	low	low	low	low	low	moderate
Zhao et al., 2017 [24]	low	moderate [c]	low	low	low	low	low	moderate
Zhu et al., 2017 [25]	low	low	low	low	low	low	low	low
Zhu et al., 2019 [26]	low	low	low	low	low	low	low	low

Methodological quality assessment was based on the Cochrane risk of bias tool (ROBINS-I). [a.] Although confounding factor remained, they included patients with similar baseline severity and who were treated with similar appliances. [b.] Selection of participants based on characteristics observed after start of intervention. [c.] Selection of participants was inappropriate between interventions but could be adjusted in analyses. [d.] Intended of intervention had minor different in intervention group. [e.] Loss follow up of participants could have affected the outcome. [f.] The outcome measurements and analyses are consistent with a prior plan.

Table S3. Studies excluded from meta-analysis of operation time.

Study	Detailed Illustration
Amano et al., 2017 [6] ◎	The result was significantly prolonged in the LH group for both unilateral and bilateral operation regardless of sex. In male population, unilateral operation time was longer in LH group compared with OH group (32.8 ± 9.2 min vs 23.3 ± 12.0 min; $p = 0.0006$) and bilateral operation time also showed similar result (45.5 ± 12.7 min vs 42.0 ± 19.9 min; $p < 0.0001$). In female population, unilateral operation time was longer in LH group compared with OH group (30.4 ± 9.1 min vs 17.2 ± 9.6 min; $p < 0.0001$) and bilateral operation time also showed similar result (42.2 ± 11.6 min vs 31.4 ± 9.2 min; $p < 0.0001$).
Chong et al., 2019 [8] △	After adjustment for the patient's baseline characteristics and surgeon, and compared with open unilateral, the incision time was longer for open+explore and open bilateral ($p < 0.0001$), slightly shorter for laparoscopic unilateral ($p = 0.01$), and about the same for laparoscopic bilateral ($p = 0.96$).
Miyake et al., 2016 [15] △	LH group showed a significant shorter operation time compared with OH group in both unilateral ($p < 0.05$) and bilateral hernia ($p < 0.01$).
Miyano et al., 2019 [16] △	The unilateral hernia and two bilateral cases were combined and LH group showed a significant shorter operation time compared with OH group ($p < 0.05$).
Saka et al., 2014 [18] ◎	The operation time was compared except for the case with an additional operation. In male population, unilateral operation time was no difference between LH and OH groups (36.7 ± 7.7 min vs 37.7±12.6 min; $p = 0.985$) and bilateral operation time showed shorter in LH group compared with OH group (46.8 ± 9.9 min vs 83.8 ± 31.6 min; $p < 0.01$). In female population, unilateral operation was longer in LH group compared with OH group (37.2 ± 8.0 min vs 29.0 ± 13.0 min; $p < 0.01$) and no bilateral open hernia repair was reported.

◎: lack exact number of patient in object of operation time to add in meta-analysis. △: lack exact data (mean or standard deviation) of operation time to add in meta-analysis.

References

1. Gause, C.D.; Casamassima, M.G.S.; Yang, J.; Hsiung, G.; Rhee, D.; Salazar, J.H.; Abdullah, F.; Lukish, J.; Colombani, P.; Chandler, N.M.; et al. Laparoscopic versus open inguinal hernia repair in children ≤ 3: A randomized controlled trial. *Pediatr. Surg. Int.* **2017**, *33*, 367–376.
2. Igwe, A.O.; Talabi, A.O.; Adisa, A.O.; Adumah, C.C.; Ogundele, I.O.; Sowande, O.A.; Adejuyigbe, O. Comparative Study of Laparoscopic and Open Inguinal Herniotomy in Children in Ile Ife, Nigeria: A Prospective Randomized Trial. *J. Laparoendosc. Adv. Surg. Tech.* **2019**, *29*, 1609–1615. https://doi.org/10.1089/lap.2019.0354.
3. Jukić, M.; Pogorelić, Z.; Šupe-Domić, D.; Jerončić, A. Comparison of inflammatory stress response between laparoscopic and open approach for pediatric inguinal hernia repair in children. *Surg. Endosc.* **2019**, *33*, 3243–3250. https://doi.org/10.1007/s00464-018-06611-y.
4. Shalaby, R.; Ibrahem, R.; Shahin, M.; Yehya, A.; Abdalrazek, M.; Alsayaad, I.; Shouker, M.A. Laparoscopic Hernia Repair versus Open Herniotomy in Children: A Controlled Randomized Study. *Minim. Invasive Surg.* **2012**, *2012*, 484135. https://doi.org/10.1155/2012/484135.
5. Zhu, X.Q.; Guan, W. Laparoscopic assisted extraperitoneal hernia sac high ligation vs traditional surgery for inguinal hernia in preschool children. *World Chin. J. Dig.* **2015**, *23*, 2168–2173.
6. Amano, H.; Tanaka, Y.; Kawashima, H.; Deie, K.; Fujiogi, M.; Suzuki, K.; Morita, K.; Iwanaka, T.; Uchida, H. Comparison of single-incision laparoscopic percutaneous extraperitoneal closure (SILPEC) and open repair for pediatric inguinal hernia: A single-center retrospective cohort study of 2028 cases. *Surg. Endosc.* **2017**, *31*, 4988–4995. https://doi.org/10.1007/s00464-017-5472-6.
7. Chang, Y.-T.; Lin, J.-Y.; Lee, J.-Y.; Tsai, C.-J.; Chiu, W.-C.; Chiu, C.-S. Comparative Mid-term Results Between Inguinal Herniotomy and Single-port Laparoscopic Herniorrhaphy for Pediatric Inguinal Hernia. *Surg. Laparosc. Endosc. Percutan. Tech.* **2012**, *22*, 526–531. https://doi.org/10.1097/sle.0b013e3182680842.
8. Chong, A.J.; Fevrier, H.B.; Herrinton, L.J. Long-term follow-up of pediatric open and laparoscopic inguinal hernia repair. *J. Pediatr. Surg.* **2019**, *54*, 2138–2144. https://doi.org/10.1016/j.jpedsurg.2019.01.064.
9. Danielson, J.; Pakkasjärvi, N.; Högberg, N. Percutaneous hernia repair in children: Safe to introduce. *Scand. J. Surg.* **2020**, *110*, 380–385. https://doi.org/10.1177/1457496920918151.
10. Darmawan, K.F.; Sinclair, T.; Dunn, J.C.Y. Comparison of laparoscopic and open pediatric inguinal hernia repairs at two institutions. *Pediatr. Surg. Int.* **2018**, *34*, 1293–1298. https://doi.org/10.1007/s00383-018-4360-z.
11. Endo, M.; Watanabe, T.; Nakano, M.; Yoshida, F.; Ukiyama, E. Laparoscopic completely extraperitoneal repair of inguinal hernia in children: A single-institute experience with 1257 repairs compared with cut-down herniorrhaphy. *Surg. Endosc.* **2009**, *23*, 1706–1712. https://doi.org/10.1007/s00464-008-0300-7.
12. Kara, Ö.; Yıldız, A.; Toydemir, H.E.; Gökyiğit, F.M.; Akın, M.; Karadağ, Ç.A.; Dokucu, A.İ.; Sever, N. Does percutaneous internal ring suturing contain risk of ilioinguinal nerve entrapment? *Pediatr. Surg. Int.* **2015**, *31*, 485–491.
13. Kara, Y.A.; Yağız, B.; Balcı, Ö.; Karaman, A.; Özgüner, I.F.; Karaman, I. Comparison of Open Repair and Laparoscopic Percutaneous Internal Ring Suturing Method in Repairing Inguinal Hernia in Children. *Cureus* **2021**, *13*, e14262. https://doi.org/10.7759/cureus.14262.
14. Liu, J.; Wu, X.; Xiu, W.; Hao, X.; Zhao, J.; Wei, B.; Dong, Q. A comparative study examining laparoscopic and open inguinal hernia repair in children: A retrospective study from a single center in China. *BMC Surg.* **2020**, *20*, 1–8. https://doi.org/10.1186/s12893-020-00912-7.
15. Miyake, H.; Fukumoto, K.; Yamoto, M.; Nouso, H.; Kaneshiro, M.; Nakajima, H.; Koyama, M.; Urushihara, N. Comparison of percutaneous extraperitoneal closure (LPEC) and open repair for pediatric inguinal hernia: Experience of a single institution with over 1000 cases. *Surg. Endosc.* **2015**, *30*, 1466–1472. https://doi.org/10.1007/s00464-015-4354-z.
16. Miyano, G.; Nakamura, H.; Shibuya, S.; Ochi, T.; Yazaki, Y.; Murakami, H.; Seo, S.; Okawada, M.; Doi, T.; Koga, H.; et al. Scrotal/testicular status after repair of recent severe incarcerated inguinal hernia in male infants younger than 12 months old: Laparoscopic percutaneous extraperitoneal closure versus conventional open repair. *Asian J. Endosc. Surg.* **2019**, *12*, 446–448. https://doi.org/10.1111/ases.12680.
17. Rao, R.; Smith, M.; Markel, T.A.; Gray, B.W.; Landman, M.P. Modified percutaneous internal ring suturing with peritoneal injury in children: Matched comparison to open hernia repair. *Surg. Endosc.* **2021**, *35*, 854–859. https://doi.org/10.1007/s00464-020-07457-z.
18. Saka, R.; Okuyama, H.; Sasaki, T.; Nose, S.; Yoneyama, C. Safety and Efficacy of Laparoscopic Percutaneous Extraperitoneal Closure for Inguinal Hernias and Hydroceles in Children: A Comparison with Traditional Open Repair. *J. Laparoendosc. Adv. Surg. Tech.* **2014**, *24*, 55–58. https://doi.org/10.1089/lap.2013.0109.
19. Shibuya, S.; Imaizumi, T.; Yamada, S.; Yoshida, S.; Yamada, S.; Toba, Y.; Takahashi, T.; Miyazaki, E. Comparison of surgical outcomes between laparoscopic percutaneous extracorporeal closure (LPEC) and open repair for pediatric inguinal hernia by propensity score methods and log-rank test analysis. *Surg. Endosc.* **2021**, 1–10. https://doi.org/10.1007/s00464-021-08354-9.
20. Timberlake, M.D.; Herbst, K.W.; Rasmussen, S.; Corbett, S.T. Laparoscopic percutaneous inguinal hernia repair in children: Review of technique and comparison with open surgery. *J. Pediatr. Urol.* **2015**, *11*, 262. https://doi.org/10.1016/j.jpurol.2015.04.008.

21. Wolak, P.; Strzelecka, A.; Piotrowska, A.; Dąbrowska, K.; Wolak, P.; Piotrowska, I.; Nowak-Starz, G. The Operative Time for Unilateral Inguinal Hernia Repair in Children Performed with Percutaneous Internal Ring Suturing (PIRS) or Open Approach Method. *J. Clin. Med.* **2021**, *10*, 1293. https://doi.org/10.3390/jcm10061293.
22. Xiao, Y. Single-port laparoscopic percutaneous extraperitoneal closure for inguinal hernias repair in girls: Using an epidural needle assisted by a towel forceps. *BMC Surg.* **2020**, *20*, 139. https://doi.org/10.1186/s12893-020-00800-0.
23. Zenitani, M.; Saka, R.; Sasaki, T.; Takama, Y.; Tani, G.; Tanaka, N.; Ueno, T.; Tazuke, Y.; Oue, T.; Okuyama, H. Safety and efficacy of laparoscopic percutaneous extraperitoneal closure for inguinal hernia in infants younger than 6 months: A comparison with conventional open repair. *Asian J. Endosc. Surg.* **2019**, *12*, 439–445. https://doi.org/10.1111/ases.12676.
24. Zhao, J.; Chen, Y.; Lin, J.; Jin, Y.; Yang, H.; Wang, F.; Zhong, H.; Zhu, J. Potential value of routine contralateral patent processus vaginalis repair in children with unilateral inguinal hernia. *BJS* **2016**, *104*, 148–151. https://doi.org/10.1002/bjs.10302.
25. Zhu, L.L.; Xu, W.J.; Liu, J.B.; Huang, X.; Lv, Z.B. Comparison of laparoscopic hernia repair and open herniotomy in children: A retrospective cohort study. *Hernia* **2017**, *21*, 417–423. https://doi.org/10.1007/s10029-017-1607-x.
26. Zhu, H.; Li, J.; Peng, X.; Alganabi, M.; Zheng, S.; Shen, C.; Dong, K. Laparoscopic Percutaneous Extraperitoneal Closure of the Internal Ring in Pediatric Recurrent Inguinal Hernia. *J. Laparoendosc. Adv. Surg. Tech.* **2019**, *29*, 1297–1301. https://doi.org/10.1089/lap.2019.0119.
